# Comparing the Neural Correlates of Conscious and Unconscious Conflict Control in a Masked Stroop Priming Task

**DOI:** 10.3389/fnhum.2016.00297

**Published:** 2016-06-20

**Authors:** Jun Jiang, Kira Bailey, Ling Xiang, Li Zhang, Qinglin Zhang

**Affiliations:** ^1^Department of Basic Psychology, School of Psychology, Third Military Medical UniversityChongqing, China; ^2^Department of Psychology, Ohio Wesleyan UniversityDelaware, OH, USA; ^3^Key Laboratory of Jiangxi Province for Psychology and Cognition Science, School of Psychology, Jiangxi Normal UniversityNanchang, China; ^4^Key laboratory of Cognition and Personality, Faculty of Psychology, Southwest UniversityChongqing, China

**Keywords:** conscious, unconscious, Stroop priming, conflict control, fMRI

## Abstract

Although previous studies have suggested that conflict control can occur in the absence of consciousness, the brain mechanisms underlying unconscious and conscious conflict control remain unclear. The current study used a rapid event-related functional magnetic resonance imaging design to collect data from 24 participants while they performed a masked Stroop priming task under both conscious and unconscious conditions. The results revealed that the fronto-parietal conflict network, including medial frontal cortex (MFC), left and right dorsal lateral prefrontal cortex (DLPFC), and posterior parietal cortex (PPC), was activated by both conscious and unconscious Stroop priming, even though in MFC and left DLPFC the activations elicited by unconscious Stroop priming were smaller than conscious Stroop priming. The findings provide evidence for the existence of quantitative differences between the neural substrates of conscious and unconscious conflict control.

## Introduction

Cognitive control refers to the ability to coordinate our thoughts and actions to achieve internal goal-directed behavior, including flexibly switching between tasks/mental sets, voluntarily initiating and overcoming habitual responses, and monitoring and resolving conflict (Brass et al., 2005; Jiang et al., 2013b). One of the most widely used paradigms to study the mechanisms of cognitive control is the Stroop color-naming task (Stroop, 1935). In the classic Stroop task, participants are instructed to name the ink color of color words. On congruent trials, the meaning of the color word and the ink color are identical (e.g., the word “red” written in red ink); on incongruent trials, the meaning of color word and the ink color are different (e.g., the word “blue” written in red ink; MacDonald et al., 2000). Typically, RTs and/or error rates (ERs) are significantly higher on incongruent than congruent trials. The size of the Stroop effect can be interpreted as the amount of conflict experienced between naming the ink color and inhibiting the automatic tendency to read the word (Panadero et al., 2015).

Previous research has extensively studied the neural substrates of the Stroop effect, revealing both frontal and parietal cortices and sub-cortices are activated, including the medial frontal cortex (MFC) (e.g., anterior cingulate cortex (ACC), pre-supplementary motor cortex (pre-SMA), supplementary motor cortex (SMA)), the dorsolateral prefrontal cortex (DLPFC), the inferior frontal gyrus (IFG)/insula, superior frontal gyrus (SFG), the posterior parietal cortex (PPC; e.g., superior/inferior parietal lobule), precuneus, middle/inferior temporal cortex, and thalamas (MacDonald et al., 2000; MacLeod and MacDonald, 2000; Egner and Hirsch, 2005; van Veen and Carter, 2005; Nee et al., 2007). The resolution of conflict experienced in the Stroop has been explained by a computational model termed the conflict-monitoring hypothesis (Botvinick et al., 2001, 2004). According to this framework, the MFC/ACC acts as performance monitoring device that detects conflict and then propagates the signal to other brain regions, in particular the DLPFC, in order to recruit cognitive control and implement strategic adjustments in subsequent performance to resolve the conflict (MacDonald et al., 2000; Panadero et al., 2015). The conflict-monitoring hypothesis does not specify the role of consciousness in that cognitive control network (Mayr, 2004). That is, the role of consciousness in cognitive control is not specified (for reviews, see Desender and Van den Bussche, 2012; Kunde et al., 2012; van Gaal et al., 2012; Ansorge et al., 2014).

Traditionally, consciousness is viewed as a necessary component of cognitive control (Norman and Shallice, 1986; Dehaene and Naccache, 2001). The prefrontal cortex not only plays a key role in recruiting and implementing cognitive control (Botvinick et al., 2001, 2004), but is also associated with conscious experience (Kouider and Dehaene, 2007). Therefore, it seems likely that consciousness is tightly linked to cognitive control; however, recent studies have convincingly suggested that unconscious information can trigger some forms of cognitive control, such as task-switching (Lau and Passingham, 2007; Reuss et al., 2011) and inhibitory control (van Gaal et al., 2009, 2010b) to name a few. Nevertheless, there is still debate about whether conflict detection and subsequent control can be triggered by unconscious information (for reviews, see Desender and Van den Bussche, 2012; Ansorge et al., 2014). This question is the focus of the current study.

Recently, researchers have attempted to understand the functions and limitations of consciousness in conflict detection and control. Using various versions of masked priming tasks, previous studies showed that the speed and accuracy of behavioral responses can be modulated by prime-induced conflict, and the neural correlates of conflict can be detected regardless of prime awareness. Specifically, participants respond faster and more accurately to congruent prime-target pairs compared to incongruent pairs even if the prime was not consciously perceived (Eimer and Schlaghecken, 1998; van Gaal et al., 2010a; Desender et al., 2013; Jiang et al., 2013a; Reuss et al., 2014; Panadero et al., 2015). Using a masked flanker-like priming task, electroencephalogram (EEG) studies have shown that unconscious conflict enhances conflict-related mid-frontal N2 (Jiang et al., 2013b; Panadero et al., 2015) and the power of theta band (Jiang et al., 2015a). Functional magnetic resonance imaging (fMRI) studies have shown that unconscious conflict activates some brain structures related to conflict monitoring and control such as the ACC, SMA, the DLPFC, and the PPC (D’Ostilio and Garraux, 2011, 2012). Although unconscious conflict detection seems to be a well-established, the neural mechanisms underlying unconscious control and the extent to which they overlap with conscious control are less well-understood (for reviews, see Desender and Van den Bussche, 2012; Kunde et al., 2012; van Gaal et al., 2012; Ansorge et al., 2014). A goal of the current study is to shed some light on this question.

In the current study, we aimed to further examine brain mechanisms of unconscious and conscious conflict detection and control, and to determine the extent of the overlap in these systems by using a rapid event-related fMRI design in combination with a masked Stroop priming task. This paradigm has been validated in unconscious conflict domain studies (Merikle and Joordens, 1997; Xiang et al., 2013; Panadero et al., 2015). For example, Merikle and Joordens (1997) asked participants to name the color of target color patches that were preceded by a prime word (e.g., “red” or “green”). Meanwhile, they manipulated the presentation duration of primes so that they were either invisible (unconscious) or visible (conscious). Similar to the classic Stroop task, they found that participants responded slower on incongruent trials than on congruent trials regardless of prime visibility. We selected this paradigm because conflict in the Stroop priming task can originate from the response set and the semantic information, and is therefore more likely to reveal the nature of unconscious conflict detection and control than a masked flanker-like priming task. Both conscious and unconscious conditions were included in the current study to compare the neural substrates.

## Materials and Methods

### Participants

Twenty-four undergraduate students (10 females) aged between 19 and 24 years (*M* = 21.00, *SD* = 1.54) were recruited from campus intranet of Southwest University in China and participated for monetary compensation. All participants were right-handed, had normal or corrected-to-normal vision, were not color-blind, and had no history of head injury. The local Ethics Committee of Southwest University approved this study, and written informed consent in accordance with the Declaration of Helsinki was obtained from all participants after the explanation of the experimental protocol.

### Apparatus and Stimuli

**Figure 1:**
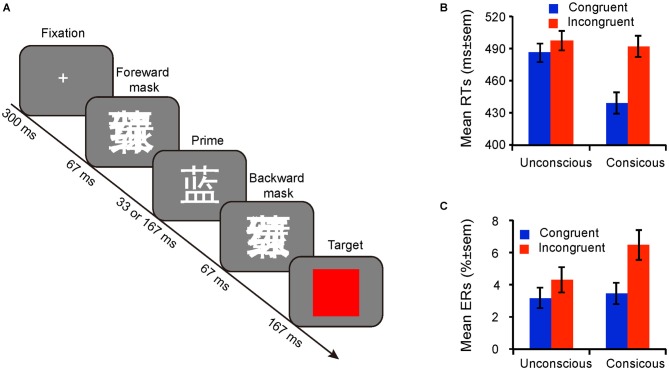
**Experimental design and behavioral results. (A)** Schematic representation of the experimental procedure and event timing. **(B)** Mean response times and **(C)** Mean error rates for congruent and incongruent trials in both conscious and unconscious conditions. Error bars represent the standard error of the mean (± SEM).

All stimuli were presented against a gray (RGB: 128, 128, 128) background at the center of a 17″ Lenovo CRT monitor (frequency 60 Hz, resolution 1024 × 768, framerate about 16.7 ms) with the E-prime 2.0 Software package (Psychology Software Tools, Pittsburgh, PA, USA). Participants performed a masked Stroop priming task, in which the primes were four white ink Chinese color words (“red”, “yellow”, ”blue”, ”green”) appearing in Song font in 36 point size, extending a visual angle of 0.96° × 1.05°. The masks were made by first overlapping the four color words, then one of them were randomly selected and inverted, and finally enlarge it to 1.1× as large as the prime (visual angle: 1.06° × 1.16°). The targets were four patches colored red, yellow, blue, or green the same size as the masks (Figure 1A).

### Design and Procedures

A rapid event-related design was used in the fMRI experimental task, in which each trial consisted of the following: a fixation point (300 ms), a forward mask (67 ms), a prime for 33 (unconscious) or 167 ms (conscious), a backward mask (identical to forward mask; 67 ms), and the target (167 ms; see Figure 1A). The trial sequences were optimized by optseq2^1^ with an average trial time of 4 s (ranging from 2 s to 16 s; van Gaal et al., 2010b). In half of the trials the prime was strongly masked, and in the other half they were weakly masked. Participants were instructed to ignore the prime word and to respond as quickly and accurately as possible to the color patch. Specifically, one half of them were asked to press “1” with the left index finger if the color of target were red or yellow, otherwise the color of target were blue or green they asked to press “2” with the right index finger. The stimulus-response mapping was counterbalanced across participants. There were 5 sessions of 128 trials each, and the unconscious and conscious trials were mixed in each session with the ratio of congruent and incongruent trials 1:1. Therefore, there were two factors: Consciousness (unconscious, conscious) and Congruency (congruent, incongruent).

Before the fMRI experiment, participants completed 48 practice trials outside of the scanner with performance feedback (mean RT and percentage correct) after each trial. After the fMRI experiment, participants performed a four-alternative forced choice (4AF) discrimination task (256 trials, the ratio of conscious and unconscious trials were 1:1) outside of the scanner to test the visibility of the primes. The timing was the same as the fMRI experiment with the following exceptions: (1) participants responded to the primes by pressing a key to indicate the word meaning (i.e., “D” = “red”, “F” = “yellow”, “J” = “blue”, “K” = “green”); (2) a screen was inserted after the target on each trial to ask the participants to discriminate the meaning of the prime. In this task, the participants were told that there was no time pressure and that we just care about the accuracy. Before the test trials, participants practiced with 48 trials to master the stimulus-response mapping rule.

### Behavioral Data Analysis

Incorrect, miss, and correct trials with an *RT* < 200 or >1000 ms were excluded from further analyses (about 4.34%). Reaction times (RTs) and ERs of conscious and unconscious trials were separately submitted to a one-way repeated measures analysis of variance (ANOVA) with congruency (congruent, incongruent) as the within-subject factor to test the Stroop priming effect. A one sample *t*-test on discrimination accuracy was used to analyze prime visibility. A two-tailed significance level of 0.05 was used for all behavioral tests.

### fMRI Images Acquisition

The images were acquired with a Siemens 3.0 T scanner (Siemens Magnetom Trio Tim, Erlangen, Germany). The T2*-weighted gradient echo planar imaging (EPI) sequences was employed to collect 266 volumes per run (repetition time (TR) = 2000 ms; echo time (TE) = 30 ms; field of view (FOV) = 220 × 220 mm^2^; and flip angle = 90°; matrix size = 64 × 64). Each volumes includes 32 interleaved slices with 3 mm thickness and 1 mm interslice gap. High-resolution T1-weighted images of each participant were also acquired, which consists of 176 slices with 1 mm thickness (*TR* = 2000 ms; *TE* = 2.52 ms; flip angle = 9°; FOV = 256 × 256 mm^2^ ; voxel size = 1 × 1 × 1 mm). All stimuli were presented on a back-projection screen, which was viewed via a mirror system attached to the scanner headcoil. The fMRI scanning session consisted of 5 sessions and 266 volumes were collected in each session.

### fMRI Data Analyses

Statistical Parametric Mapping (SPM8, Wellcome Department of Cognitive Neurology, London, UK) software was used to preprocess and analyze the MRI data in Matlab (Matlab 2009a, Math works, Natick, MA, USA). Functional data were first temporally and then spatially realigned to correct for the time difference and small head movements during image acquisition, respectively. Next, the functional data were spatially normalized to Montreal Neurological Institute (MNI) space based on the functional EPI template implemented in SPM8 and simultaneously resampled to 3 × 3 × 3 mm^3^ resolution. Finally, the functional data were smoothed with an 8 mm full-width-at-half-maximum (FWHM) Gaussian kernel. Given the T1 saturation effects, five volumes acquired before stimulus presentation were discarded.

After the preprocessing, five regressors (conscious congruent [CC], conscious incongruent [CI], unconscious congruent [UC], unconscious incongruent [UI], and error response) time-locked to target onset were modeled on the functional data of each participant and each run to create the design matrix using general linear model. To correct the head movement related artifacts, six parameters of head movements were also included in the model as a covariate of no interest. Each regressor was convolved with the hemodynamic response function. To eliminate low-frequency noise, a high-pass filter (σ = 128 s) was applied. Using one sample *t*-tests, six first-level contrasts were created. Specifically, we separately compared CC, CI, UC and UI with null events, and to examine the neural activity of typical Stroop priming effect under each conscious level, we also separately created another two contrasts (conscious Stroop priming effect: CI > CC and unconscious Stroop priming effect: UI > UC). Then, the generated contrast images were submitted to group-level random effect analyses to estimate error variance across individuals. To directly compare brain activation of Stroop priming effects between conscious and unconscious conditions, the group-level full factorial design comprised the factors of consciousness (conscious vs. unconscious) and congruency (congruent vs. incongruent). To include only gray matter (GM) voxels of interest and to reduce the number of multiple comparisons in the group-level analyses, we used a GM mask, which has a higher probability than either white matter (WM) and cerebrospinal fluid (CSF) [(GM > WM)∩(GM > CSF)] (Villain et al., 2010). Moreover, to correct for multiple comparisons, 1000 Monte Carlo simulations were calculated using AlphaSim implemented in AFNI at the whole-brain threshold of uncorrected *p* < 0.001 with a cluster connection radius in 5 mm and an estimate of spatial smoothness of the residual (Gaussian filter width: FWHM_X_ = 12.17 mm, FWHM_y_ = 12.26 mm, FWHM_z_ = 12.00 mm, which is estimated by first averaging the smoothness of the SPM first level GLM residuals (saved in SPM.xVol.FWHM) across subjects, and then multiplying the size of voxel, for more details please see the manual of the SPM8w toolbox^2^). Using this procedure at a threshold of *p* < 0.001 resulted in a cluster threshold of 36 contiguous voxels. The anatomical labels of peak and subpeaks voxel of significant clusters were determined using the Talairach atlas (tdclient; Denny et al., 2014), which implemented in the NeuroElf toolbox (V1.0^3^). The reported coordinates are in MNI space.

In order to further explore the activation difference between unconscious and conscious Stroop priming effects, and to avoid Type I error, we also conducted regions of interests (ROIs) analyses. First, five ROIs (MFC, left and right DLPFC, left and right PPC) were defined by intersecting the significant activation clusters of the main effect of congruency and the anatomy in Talairach atlas, and then the mean beta values within each ROI under CC, CI, UC and UI conditions were extracted by using Marsbar toolbox (Brett et al., 2002), which were separately submitted to a two-way ANOVA with consciousness and congruency as within-subject factors.

## Results

### Discrimination Results

The results of the 4AFC discrimination task showed that accuracy was not higher than chance level (25%) in the strongly masked condition (*M* = 26.54%, *t*_(23)_ = 1.21, *p* > 0.05), while accuracy was far greater than chance level in the weakly masked condition (*M* = 86.21%, *t*_(23)_ = 60.23, *p* < 0.001); the difference between the conditions was significant (*t*_(23)_ = 35.52, *p* < 0.001). Therefore, awareness of primes was well manipulated in the current study.

### Behavioral Results

As illustrated in Figures 1B,C, participants responded faster (unconscious: *F*_(1,23)_ = 27.81, *p* < 0.001, *η*^2^ = 0.55; conscious: *F*_(1,23)_ = 263.13, *p* < 0.001, *η*^2^ = 0.92) and committed fewer errors (unconscious: *F*_(1,23)_ = 11.16, *p* = 0.003, *η*^2^ = 0.33; conscious: *F*_(1,23)_ = 21.23, *p* < 0.001, *η*^2^ = 0.48) to congruent trials than to incongruent trials, indicating that the Stroop priming effect occurred irrespective of prime consciousness. A two-way ANOVA with consciousness and congruency as within-subject factors showed that the two-way interaction was significant (RTs: *F*_(1,23)_ = 160.91, *p* < 0.001, *η*^2^ = 0.88; ERs: *F*_(1,23)_ = 10.40, *p* = 0.004, *η*^2^ = 0.31), meaning that the Stroop priming effect in conscious trials (RTs: *M* = 53 ms; *SE* = 3.34 ; ERs:* M* = 3.00%; *SE* = 0.67%) was far larger than in unconscious trials (RTs: *M* = 11 ms; *SE* = 2.10; ERs:* M* = 1.13%; *SE* = 0.34%), both for RTs and ERs.

### fMRI Results

**Figure 2:**
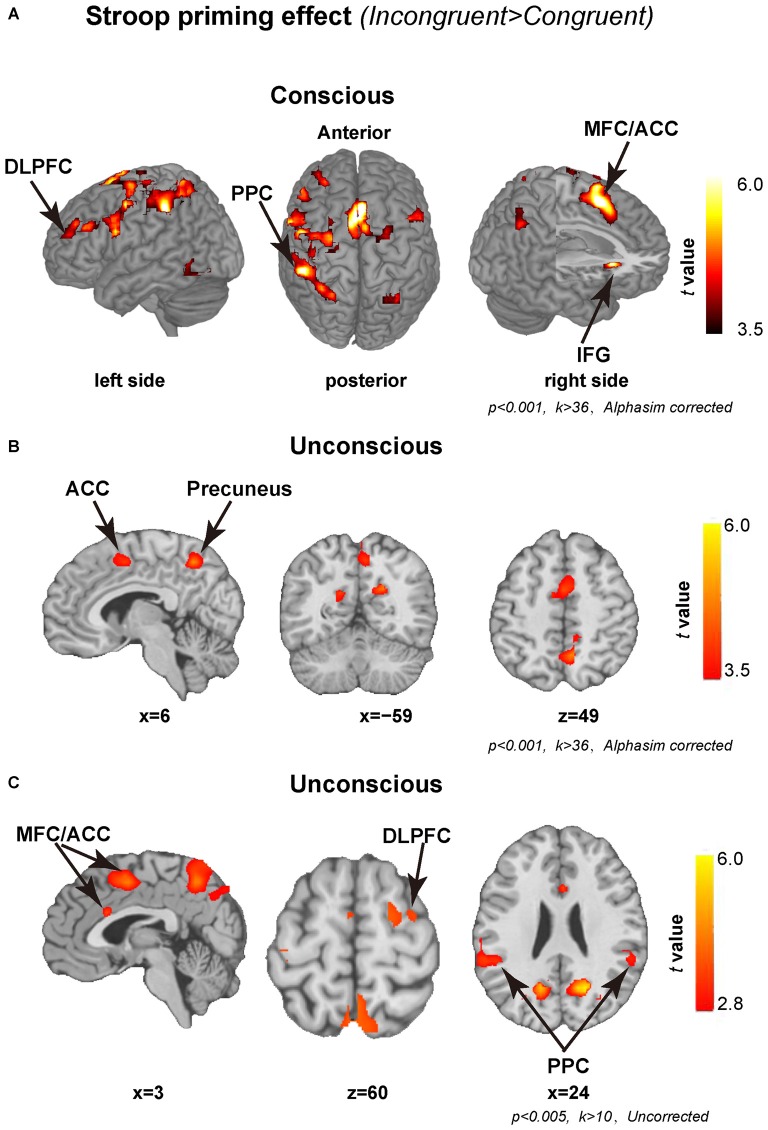
**Brain regions showing significant activation by conscious and unconscious Stroop priming effects. (A)** Regions showing significant activation by conscious Stroop priming effect. **(B)** Regions showing significant activation by unconscious Stroop priming effect. The statistical thresholds were set at *p* < 0.001 with a minimum cluster size of 36 voxels using the AlphaSim Monte Carlo simulation in **(A,B)**. **(C)** Brain regions showing significant activation by unconscious Stroop priming effects. The statistical thresholds were set at *p* < 0.005 with a minimum cluster size of 10 voxels, uncorrected. DLPFC, dorsal lateral prefrontal cortex; PPC, posterior parietal cortex; ACC, anterior cingulate cortex; MFC, medial frontal cortex.

Figure 2 shows the activation pattern elicited by conscious and unconscious Stroop priming effects. In line with previous studies using the typical Stroop task, the conscious Stroop priming effect activated the fronto-parietal conflict control network (MacDonald et al., 2000; MacLeod and MacDonald, 2000; Kerns et al., 2004; Egner and Hirsch, 2005; Nee et al., 2007), including the MFC/ACC, DLPFC, pre-SMA, IFG, SFG, insula, PPC (such as inferior parietal gyrus, superior parietal gyrus) and precuneus (Figure 2A). In contrast, only part of this network was activated by the unconscious Stroop priming effect, including the MFC and precuneus (Figure 2B). Detailed patterns of activation by unconscious and conscious Stroop priming effects are shown in Table 1. The conscious and unconscious contrasts showed that a cluster in MFC (68 of 77 unconsciously activated voxels overlapped with consciously activated voxels, 88.31% overlap, see “Supplementary Figure 1”) activated by Stroop priming effects very similar. The other clusters nearly not overlap.

**Table 1:** **Contrasts (Incongruent > congruent) of brain activations (BA) for both conscious and unconscious Stroop priming effect**.

Consciousness	Regions	Side	BA	*x*	*y*	*z*	*t*	*k*
**Conscious**	Superior frontal/Cingulate gyrus	L/R	6/8/24/32	8	18	48	7.36	679
	middle/superior/inferior/frontal/cingulate/precentral gyrus	L	9/40/3/44/6/46	−45	−36	42	6.60	1108
	Insula	R	13	33	21	6	6.07	77
	Middle/Inferior frontal gyrus	R	9	45	9	33	5.04	141
	Middle/Superior frontal gyrus	L	10	−27	42	27	4.85	93
	Superior parietal lobule	R	7	36	−63	45	4.60	80
	Fusiform/middle temporal gyrus	L	37	−42	−57	−12	4.28	70
	Middle frontal gyrus	R	9	45	33	30	4.19	37
**Unconscious**	Precuneus	R	31	21	−63	24	6.32	62
	Inferior occipital gyrus	L	17	−24	−96	−9	5.88	85
	Precuneus/cingulate gyrus	R	7/31	9	−54	48	5.87	88
	Precuneus	L	31	−15	−63	24	5.45	53
	Cingulate/Medial frontal gyrus	L/R	6/24	−9	−3	51	4.84	77

*Note: with magnitude and spatial extent thresholds at p < 0.001 and k > 36 voxels, AlphaSim corrected. k-values and t-values are reported for peak voxels of each cluster. The reported coordinates are in MNI space*.

From Figure 2B we can see that some key nodes predicted by the conflict monitoring model, such as DLPFC and MFC/ACC, were not activated by unconscious Stroop priming conflict at the above mentioned statistical threshold level. Given the fact that, subliminal priming effects are very small and accordingly difficult to detect, the activation threshold and multiple comparison correction method used in the current study may be too stringent. Similar to previous studies (Kouider and Dehaene, 2007; Kouider et al., 2010; van Gaal et al., 2010b; D’Ostilio and Garraux, 2012), we did an exploratory analysis on unconscious trials with the criteria that any results surviving a peak threshold of *p* < 0.005 uncorrected and a cluster threshold of 10 contiguous voxels in these regions were regarded as significant (D’Ostilio and Garraux, 2012). As shown in Figure 2C and “Supplementary Table 1”, this analysis revealed additional regions of the fronto-parietal conflict control network were activated by the unconscious Stroop priming effect, such as right DLPFC, left and right PPC and the MFC/ACC.

**Figure 3:**
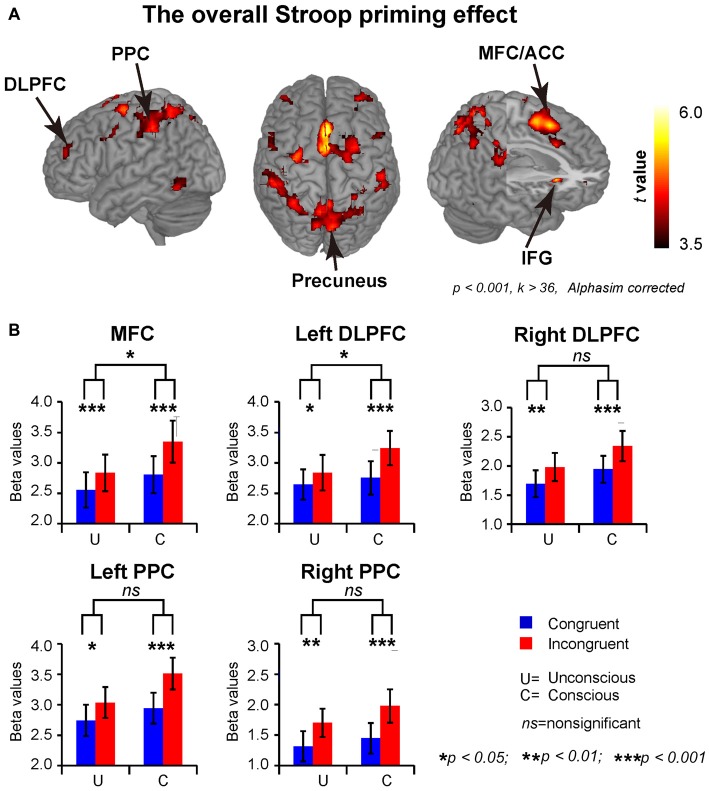
**Brain regions showing significant activation of the overall Stroop priming effects. (A)** The overall Stroop priming effects (Incongruent > congruent). The statistical thresholds were set at *p* < 0.001 with a minimum cluster size of 36 voxels using the AlphaSim Monte Carlo simulation. **(B)** The ROI analyses results. The Stroop prime effects were significant at MFC, left and right DLPFC, and the left and right PPC in both unconscious and conscious conditions, though the activations in conscious condition were stronger at MFC and left DLPFC than in unconscious condition. MFC, medial frontal cortex; DLPFC, dorsal lateral prefrontal cortex; PPC, posterior parietal cortex.

To investigate the difference between conscious and unconscious conflict control networks, we conducted a group-level full factorial design analysis. The results showed that the interaction between consciousness and congruency was not significant. The main effect of congruency suggested that the overall Stroop priming effect activated the frontal-parietal conflict control network, including the MFC/ACC, DLPFC, PPC, SFG, IFG and insula (see Figure 3A and “Supplementary Table 2”).

As illustrated in Figure 3B, the ANOVAs on the ROI data showed that the conscious Stroop priming conflicts (MFC: *M* = 0.54, *SE* = 0.08, *t*_(23)_ = 6.83, *p* < 0.001; left DLPFC:* M* = 0.49, *SE* = 0.09, *t*_(23)_ = 5.14, *p* < 0.001) were larger than unconscious Stroop priming conflicts (MFC: *M* = 0.28, *SE* = 0.08, *t*_(23)_ = 3.78, *p* < 0.001; left DLPFC:* M* = 0.19, *SE* = 0.08, *t*_(23)_ = 2.47, *p* = 0.021) at both MFC and left DLPFC, as indexed by the significant two-way interaction between the factors of consciousness and congruency (MFC: *F*_(1,23)_ = 5.51, *p* = 0.028, *η*^2^ = 0.19; left DLPFC: *F*_(1,23)_ = 4.83, *p* = 0.038, *η*^2^ = 0.17), whereas the interactions at right DLPFC (*F* < 1), left PPC (*F*_(1,23)_ = 1.20, *p* = 0.175, *η*^2^ = 0.08) and right PPC (*F* < 1) were not significant; the conscious (right DLPFC: *M* = 0.40, *SE* = 0.09, *t*_(23)_ = 4.25, *p* < 0.001; left PPC: *M* = 0.57, *SE* = 0.11, *t*_(23)_ = 5.01, *p* < 0.001; right PPC: *M* = 0.53, *SE* = 0.12, *t*_(23)_ = 4.62, *p* < 0.001) and unconscious (right DLPFC: *M* = 0.29, *SE* = 0.08, *t*_(23)_ = 3.54, *p* = 0.002; left PPC: *M* = 0.29, *SE* = 0.12, *t*_(23)_ = 2.41, *p* = 0.024; right PPC: *M* = 0.39, *SE* = 0.13, *t*_(23)_ = 2.92, *p* = 0.008) Stroop priming conflict effects were both significant at those areas.

## Discussion

In the current study, we explored the neural substrates of conflict detection and control at different levels of conflict awareness by using a masked Stroop priming task. To our knowledge, this is the first study to reveal differences in the neural correlates of the Stroop priming effect at different levels of conflict awareness. Behaviorally, consistent with previous studies using the typical Stroop task (MacDonald et al., 2000; MacLeod and MacDonald, 2000; Egner and Hirsch, 2005; van Veen and Carter, 2005), the results showed the Stroop priming effect regardless of the consciousness of primes, namely, participants responded slower and less accurately to incongruent trials than to congruent trials under both conscious and unconscious conditions. On the neural level, the fMRI results showed that the unconscious and conscious Stroop priming effects activated the fronto-parietal conflict control network, including the MFC/ACC, DLPFC and PPC. Moreover, while most of the blood oxygenation level dependent (BOLD) signals evoked by the conscious and unconscious versions of conflict control were very similar, the neural activity evoked by unconscious Stroop priming was far weaker and the range was smaller than conscious Stroop priming.

According to the conflict monitoring hypothesis, the ACC is associated with conflict detection, which has been confirmed in various conflict involvement tasks (MacDonald et al., 2000; MacLeod and MacDonald, 2000; Egner and Hirsch, 2005; van Veen and Carter, 2005). Consistent with the conflict monitoring theory and previous work, the current study found the ACC activated by both conscious and unconscious Stroop priming conflict. Recently, combining a Flanker-like task and fMRI methods, D’Ostilio and Garraux (2012) found that the ACC was involved in response conflict detection even when the conflict was not consciously perceived. In other neuroimaging studies, researchers have found that the ACC is activated when the stimuli presentation sequence violated implicit probabilistic learning rules (Ursu et al., 2009) or implicit expectations (Rose et al., 2005), even when the participants are not consciously aware of the rules or expectations. In an event related potential study, Xiang et al. (2013) found that the conscious and unconscious Stroop priming conflict elicited the N450, a component assumed to reflect conflict detection, when participants performed a Stroop-like task similar to the task used in the present study. Further, source location analyses showed that the N450 component likely originates in ACC. Similarly, we have found that medial frontal theta-band power increases in incongruent trials compared to congruent trials, irrespective of conflict awareness, during a masked Flanker-like task, an effect that has also been localized to the ACC (Jiang et al., 2015a). Those studies suggest that ACC activation is independent of consciousness. Nevertheless, it is worth noting that there are a few conflicting findings (Dehaene et al., 2003; Praamstra and Seiss, 2005). For instance, Dehaene et al. (2003) only found significant ACC BOLD activity changes for conscious conflict but not for unconscious conflict in a masked numerical magnitude judgment task, even though they found a behavioral conflict effect independent of conflict awareness. However, there are alternative interpretations of this negative result: first, given conscious conflict effects were nearly twice as large as unconscious conflict effects, it may have been difficult to find statistically significant ACC activation for unconscious conflict at the thresholds used in the study (Mayr, 2004); second, there was no baseline condition to compare the activation of congruent and incongruent conditions (D’Ostilio and Garraux, 2012); ACC activation is not only found on incongruent trials but sometimes on congruent trials when compared with a baseline condition (neutral trials; Roelofs et al., 2006; Ursu et al., 2009). In the ROI analyses, we found that the activation of ACC elicited by unconscious Stroop conflict was smaller relative to conscious Stroop conflict, possibly because the intensity of conflict elicited was smaller under the unconscious condition than under the conscious condition.

In the current study, the left and right DLPFC was activated by conscious Stroop conflict, which is consistent with previous fMRI (Kerns, 2006; Kim et al., 2014; Salami et al., 2014) and electrophysiological studies (Cavanagh et al., 2009; Nigbur et al., 2012; Cohen and van Gaal, 2013). On the other hand, there was no significant activation of left or right DLPFC for unconscious Stroop conflict if a stringent statistical criterion was adopted (*p* < 0.001, *k* > 36, Alphasim corrected). While a slightly relaxed statistical criterion was adopted (*p* < 0.005, *k* > 10, uncorrected) based on previous studies, the right DLPFC was activated further indicating that the conflict was detected in bilateral DLPFC, even though the unconscious Stroop conflict was far smaller than conscious Stroop conflict. These results are consistent with previous neuroimaging studies of unconscious cognitive control that have shown activity in the DLPFC is modulated by unconscious conflict (D’Ostilio and Garraux, 2011, 2012; van Gaal et al., 2011).

In line with previous studies using the classic Stroop task (van Veen and Carter, 2005; Chen et al., 2013) or a Stroop-like task (Kim et al., 2010), the current study found that in addition to frontal regions, posterior parietal areas, such as PPC (BA 7/40; Kim et al., 2010), were also involved in conscious and unconscious conflict resolution. The ROI analyses showed that there was no difference in activation levels between unconscious and conscious Stroop conflict. The PPC may adopt the same strength of control to resolve conflict, even if the unconscious conflict is smaller than conscious conflict. As a part of fronto-parietal cognitive control or attentional network, the PPC is generally assumed to be associated with task-relevant stimulus-response mappings, stimuli selection, and response transformations or to be involved in motor preparation and visuomotor integration during response execution (Andersen and Buneo, 2002; van Veen and Carter, 2005; Culham et al., 2006). The common activation in PPC may indicate that both conscious and unconscious conflict control need top down modulation from prefrontal regions to posterior parietal regions during attentional processing or response selection (van Veen and Carter, 2005; Kim et al., 2010; Chen et al., 2013). In our recent EEG study, we revealed that the Stroop priming effect contains both response and semantic/stimulus conflict regardless of the level of conflict awareness (Jiang et al., 2015b). Moreover, previous study using the classic Stroop task demonstrate activation of PPC by both semantic and response conflict (van Veen and Carter, 2005; Chen et al., 2013). We did not distinguish between semantic/stimulus and response conflict in the current study, but based on the previous literature it is like that the PPC activation found here is also elicited by both semantic and response conflict.

Whether the difference between conscious and unconscious cognitive control is quantitative or qualitative has remained an open question in the literature on unconscious processing. Horga and Maia (2012) proposed the difference is quantitative, holding that unconscious and conscious cognitive control might share the same neural substrates, and that the distinction between the two likely originates from the quality of representation. Other works have found evidence of qualitative differences between conscious and unconscious conflict control (Merikle and Joordens, 1997; Daza et al., 2002). The intensity and activation range difference between the unconscious and conscious Stroop priming effects in the current study appear to support a quantitative difference, but not necessarily a qualitative one. The current result did not indicate any area of the fronto-parietal conflict control network specific to unconscious and conscious Stroop conflict. In addition, the ROI analyses only revealed quantitative differences in MFC/ACC and left DLPFC.

In conclusion, this work suggests that cognitive control can occur independent of conscious awareness of the conflict, rather than being tightly linked to it. Moreover, the findings support the existence of quantitative differences in the neural substrates of conscious and unconscious conflict control. Our findings extend our understanding of the mechanisms of cognitive control and on the limitations and function of consciousness.

## Author Contributions

JJ and QZ designed the experiment and analyzed the data, JJ, KB, LX and LZ wrote the article.

## Conflict of Interest Statement

The authors declare that the research was conducted in the absence of any commercial or financial relationships that could be construed as a potential conflict of interest.

## Footnotes

^1^http://surfer.nmr.mgh.harvard.edu/optseq/

^2^https://github.com/ddwagner/SPM8w

^3^http://neuroelf.net/
