# Supplementary material for: Comparing the Neural Correlates of Conscious and Unconscious Conflict Control in a Masked Stroop Priming Task
Source: Front Hum Neurosci. 2016 Jun 20;10:297. doi: 10.3389/fnhum.2016.00297 (PMC4913088; doi:10.3389/fnhum.2016.00297)
Supplement: Supplementary file 1 [file DataSheet_1.pdf]

Supplementary Material

Comparing the Neural Correlates of Conscious and Unconscious Conflict Control in a Masked Stroop Priming Task

Jun **Jiang**[*], Kira **Bailey**, Ling **Xiang**, Li, **Zhang**, Qinglin **Zhang**

*** Correspondence:** Corresponding Author: jun.qq.jiang@gmail.com

1 Supplementary Figure and Table

1.1 Supplementary Table

Supplementary Table S1 The brain activation elicited by unconscious Stroop priming effect

**Regions**	**Side**	**BA**	**x**	**y**	**z**	**t**	***k***
Precuneus	R	31	21	-63	24	6.32	539
Precuneus	R	7	9	-54	48	5.87	
Precuneus	R	7	12	-78	51	4.19	
Cingulate Gyrus	R	31	9	-39	45	4.12	
Precuneus	L	7	-3	-63	63	3.73	
Precuneus	L	7	-9	-54	48	3.63	
Precuneus	L	7	-9	-81	48	3.31	
Posterior Cingulate	R	30	18	-54	12	3.24	
Superior Parietal Lobule	R	7	18	-63	66	3.18	
Precuneus	L	7	-3	-75	36	2.98	
Inferior Occipital Gyrus	L	17	-24	-96	-9	5.88	149
Precuneus	L	31	-15	-63	24	5.45	171
Lingual Gyrus	L	18	-18	-72	6	4.71	
Parahippocampal Gyrus	L	19	-21	-54	0	3.38	
Cingulate Gyrus	L	24	-9	-3	51	4.84	177
Medial Frontal Gyrus	L	6	0	0	48	4.71	
Cingulate Gyrus	R	24	9	3	48	4.40	
Inferior Occipital Gyrus	R	18	39	-87	-6	4.30	67
Fusiform Gyrus	R	19	27	-81	-12	3.37	
Middle Occipital Gyrus	R	18	33	-84	3	2.99	
Postcentral Gyrus	R	2	60	-30	42	4.16	88
Postcentral Gyrus	R	2	60	-30	42	4.16	
Postcentral Gyrus	R	2	57	-21	54	3.69	

Inferior Frontal Gyrus	R	46	48	39	9	4.16	16
Declive	R		45	-54	-18	4.15	94
Fusiform Gyrus	R	19	45	-66	-6	3.30	
Middle Temporal Gyrus	R	39	33	-66	27	4.01	36
Superior Parietal Lobule	R	7	27	-63	42	3.83	
Inferior Frontal Gyrus	R	9	60	12	33	3.89	22
Inferior Frontal Gyrus	R	9	45	3	33	3.50	
Inferior Parietal Lobule	L	40	-60	-42	21	3.87	129
Postcentral Gyrus	L	40	-63	-24	18	3.57	
Inferior Parietal Lobule	L	40	-51	-42	24	3.56	
Superior Temporal Gyrus	L	22	-66	-48	9	3.27	
Superior Temporal Gyrus	L	42	-69	-21	9	3.18	
Sub-Gyral	R	6	24	0	57	3.85	83
Sub-Gyral	R	6	24	0	57	3.85	
Precentral Gyrus	R	6	27	-12	72	3.57	
Superior Frontal Gyrus	R	6	12	6	69	3.46	
Middle Frontal Gyrus	R	6	36	0	60	3.05	12
Anterior Cingulate	R	24	3	18	24	3.85	
Superior Occipital Gyrus	L	19	-30	-69	27	3.67	15
Superior Temporal Gyrus	R	13	57	-39	21	3.46	41
Superior Temporal Gyrus	R	22	57	0	-3	3.43	57
Superior Temporal Gyrus	R	22	57	9	0	3.34	
Superior Temporal Gyrus	R	38	57	9	-9	3.34	
Precentral Gyrus	R	44	60	15	9	3.20	
Declive	L		-33	-57	-18	3.37	46
Fusiform Gyrus	L	19	-39	-66	-9	3.24	
Inferior Parietal Lobule	R	40	36	-42	45	3.29	17
Postcentral Gyrus	L	3	-39	-24	63	3.15	10

Note: with magnitude and spatial extent thresholds at p < 0.001 and k > 36 voxels, AlphaSim corrected. k-values and t-values are reported for peak voxels of each cluster. Additional subpeaks (no k-values were given in this table) within larger clusters are included for descriptive purposes. The star on k means this cluster pass the AlphaSim correction with k>80 voxels. p<0.005. The reported coordinates are in MNI space.

Supplementary Table S2 The brain activation of the main effect of congruency

Regions	Side	BA	x	y	z	t	*k*
Superior/Medial/Middle Frontal/Anterior Cingluate/Cingulate/Gyrus	L/R	8/6/24/32	3	18	48	5.68	1150
Declive /Middle Temporal Gyrus	R		45	-54	-18	5.40	175
Insula	R	13	33	18	6	5.33	72
Middle Frontal Gyrus/ Superior/Inferior Parietal Lobule/Precuneus	L/R	6/40/22/7	-27	-9	60	4.91	2214
Insula	L	13	-36	15	6	4.55	49
Middle/Superior Frontal Gyrus	R	10/9	36	45	15	4.45	240
Fusiform/ Middle Temporal Gyrus/ Declive /Culmen	L	37	-51	-60	-15	4.32	140
Inferior Occipital Gyrus/Declive	R	18	36	-87	-9	4.26	96
Middle Frontal/PrecentraGyrus	L	10/9/6/46	-36	48	18	4.23	241
Middle Frontal Gyrus	R	9	48	9	33	4.09	87
Posterior Cingulate/ Cuneus/Precuneus	R	30/18/31/19	15	-57	9	4.00	261

Note: with magnitude and spatial extent thresholds at $p < 0.001$ and $k > 36$ voxels, AlphaSim corrected. k-values and t-values are reported for peak voxels of each cluster. The reported coordinates are in MNI space. The reported coordinates are in MNI space.

1.2 Supplementary Figure

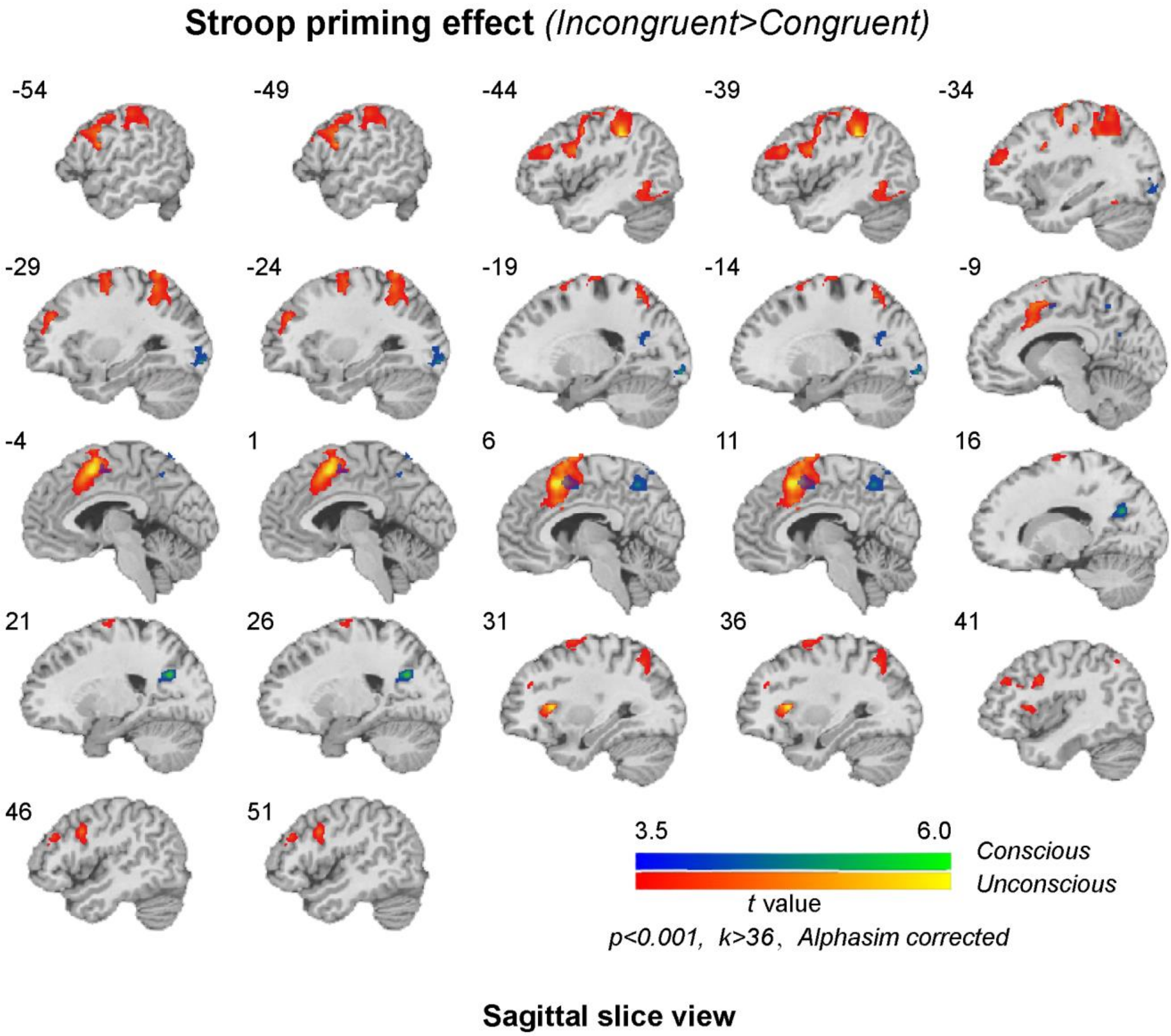

Supplementary Figure S1. The sagittal slice view of brain regions showing significant activation by conscious and unconscious Stroop priming effects. The statistical thresholds were set at $p < 0.001$ with a minimum cluster size of 53 voxels using the AlphaSim Monte Carlo simulation. This figure shows that the brain activations elicited by conscious and unconscious Stroop priming effects do not completely overlap.
